# Drug Prevention and Control of Ventilator-Associated Pneumonia

**DOI:** 10.3389/fphar.2019.00298

**Published:** 2019-03-28

**Authors:** Xinming Xie, Jun Lyu, Tafseel Hussain, Manxiang Li

**Affiliations:** ^1^Department of Respiratory and Critical Care Medicine, The First Affiliated Hospital, Xi’an Jiaotong University, Xi’an, China; ^2^Clinical Research Center, The First Affiliated Hospital, Xi’an Jiaotong University, Xi’an, China

**Keywords:** antibiotics, monoclonal antibodies, probiotics, chlorhexidine, ventilator-associated pneumonia

## Abstract

Ventilator-associated pneumonia (VAP) is one of the most prevalent and serious complications of mechanical ventilation, which is considered a common nosocomial infection in critically ill patients. There are some great options for the prevention of VAP: (i) minimize ventilator exposure; (ii) intensive oral care; (iii) aspiration of subglottic secretions; (iv) maintain optimal positioning and encourage mobility; and (v) prophylactic probiotics. Furthermore, clinical management of VAP depends on appropriate antimicrobial therapy, which needs to be selected based on individual patient factors, such as previous antibacterial therapy, history of hospitalization or mechanical ventilation, and bacterial pathogens and antibiotic resistance patterns. In fact, antibiotic resistance has exponentially increased over the last decade, and the isolation of a multidrug-resistant (MDR) pathogen has been identified as an independent predictor of inadequate initial antibiotic therapy and which is significantly associated with increased mortality. Multiple attempts were used in the treatment of VAP, such as novel antibacterial agents, inhaled antibiotics and monoclonal antibodies. In this review, we summarize the current therapeutic options for the prevention and treatment of VAP, aiming to better management of VAP in clinical practice.

## Introduction

Ventilator-associated pneumonia (VAP) is believed to be the most commonly acquired infection in the intensive care unit (ICU) and is associated with high morbidity and mortality rates (Timsit et al., 2017a; Vandana Kalwaje and Rello, 2018). The incidence of VAP ranges from 10 to 25% of all ICU patients, the VAP-related mortality rate is between 24 and 76%, which is 6–21 times higher in the intubated patients (Dasgupta et al., 2015). VAP usually occurs 48–72 h after mechanical ventilation and is related to the increased incidence of multidrug-resistant (MDR) infections, prolonged mechanical ventilation, increased antibiotic usage, and patient stay in the hospital (Charles et al., 2014). Although prevention efforts may reduce the frequency of these infections, unfortunately, only a few preventive strategies have been demonstrated to be effective in managing VAP, while many others should be further evaluated in large randomized trials before becoming the clinical recommendations (Li Bassi et al., 2017). In addition, it’s very challenging to make the correct diagnosis of VAP in the clinical setting in the absence of a gold standard (Tejerina et al., 2010; Nair and Niederman, 2015; Timsit et al., 2017a).

A prevention policy aiming to reduce VAP remains an important element of the management for patients admitted to ICUs and requiring mechanical ventilation. The current preventive strategies for VAP are mainly directed at colonization and aspiration modification, such as avoiding intubation (Carron et al., 2013), oral care (Nicolosi et al., 2014), assessing for early weaning and mobility (Balas et al., 2014; Klompas et al., 2015), and prophylactic probiotics (Wong et al., 2015). The empiric treatment strategies of VAP should be informed by the local distribution of pathogens and their antimicrobial susceptibilities, because the pathogens responsible for VAP and the drug-resistance situation are varying from region to region (Weber et al., 2007; Chung et al., 2011; Mathai et al., 2016; Awad et al., 2018). VAP is commonly caused by *Pseudomonas aeruginosa*, *Klebsiella pneumonia*, and *Acinetobacter baumannii* globally. It is important that the antimicrobial therapy be right in the first time in VAP patients, because the pathogens associated with inappropriate initial therapy are usually antibiotic-resistant strains of these pathogens, so patients at high risk of infection with these organisms initially needed to receive a combination of agents providing a very broad spectrum of coverage (Weiner et al., 2016; Rhodes et al., 2018). This review aims to summarize the available knowledge on drug prevention and control of VAP, taking profit from the recommendations of several health organization such as the American Thoracic Society with the Infectious Disease Society of America (Kalil et al., 2016), the Centers for Disease Control and Prevention (CDC) (Tablan et al., 2004), the European Task Force on VAP (Torres and Carlet, 2001), the Society for Healthcare Epidemiology of America, and the Institute for Healthcare Improvement (Klompas et al., 2014), as well as the recently major guidelines for the management of VAP (Kalil et al., 2016; Timsit et al., 2017a; Torres et al., 2017), and we hope that our clinicians know more about the initial therapies and treatment strategies of VAP, and we can investigate and focus on the management of the disease in future.

## Definition and Diagnosis of VAP

**FIGURE 1:**
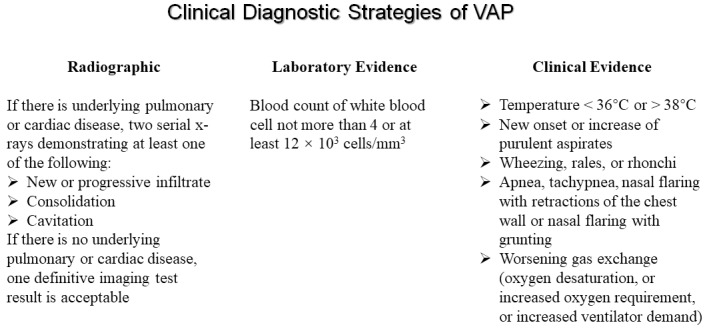
The clinical diagnostic strategies of ventilator-associated pneumonia.

Currently, there is no gold standard and valid definition for VAP, even the most widely used VAP criteria and definitions are neither sensitive nor specific. From 2013, the ventilator-associated event (VAE) was established by CDC based on a novel and multi-tiered algorithm, including definitions for a ventilator-associated condition (VAC), infection-related ventilator-associated complication (IVAC), and possible or probable VAP (PVAP). These definitions were developed in order to better capture infectious and non-infectious events in patients receiving mechanical ventilation (Magill et al., 2013). Unfortunately for the bedside practitioner, the new CDC definition for VAP has been found to have a sensitivity as low as 37% with a negative predictive value of only 84%, suggesting that VAE algorithm might not be intended for use in the clinical management of patients. In 2016, CDC reported a module about the definition of VAP is pneumonia that arises at least 48 h after endotracheal intubation (Erb et al., 2016). As shown in Figure 1, despite lacking a gold standard definition for VAP, they are still based on a combination of radiographic, laboratory, and clinical findings. Clinical suspicion of VAP in a patient is the initial part of the diagnosis, and the recommended standard diagnostic criteria for VAP is as follows: (i) radiographic (such as new or progressive and persistent infiltrates, consolidation or cavitation); (ii) laboratory evidence (such as blood count of white blood cell not more than 4 or at least 12 × 10^3^ cells/mm^3^); (iii) clinical evidence (such as temperature <36°C or >38°C, new onset or increase of purulent aspirates, wheezing, rales, rhonchi, or worsening gas exchange) (Grgurich et al., 2013; Timsit et al., 2017a; Torres et al., 2017; Metersky and Kalil, 2018; Vazquez Guillamet and Kollef, 2018).

In addition, microbiologic criteria are also an important element for VAP in a clinical context, such as positive culture results from suctioned sputum, bronchoscopy, blind bronchoalveolar lavage (BAL), or pleural fluid. Although not required to diagnose VAP, serial cultures and microscopic assessments of tracheal aspirate samples during treatment can be useful gauges of the patient’s response to antibiotics (Mariki et al., 2014; Fernandez-Barat et al., 2017). VAP can also be divided into early- and late-onset VAP. VAP developing within 4 days of admission are defined as early-onset VAP, which are usually caused by microorganisms sensitive to antibiotics. VAP occurring more than 4 days after admission are defined as late-onset VAP, which are most commonly associated with MDR pathogens and related to higher mortality than early-onset VAP (Nguile-Makao et al., 2010; Martin-Loeches et al., 2015; Torres et al., 2017).

## Causative Pathogens and Risk Factors of VAP

A lot of microbes were detected as a causative agent of VAP, the common pathogens of VAP include Gram-negative bacteria such as *P. aeruginosa*, *Escherichia coli*, *Acinetobacter species*, and *K. pneumoniae*, and Gram-positive bacteria such as *Staphylococcus aureus* (Chi et al., 2012; Ding et al., 2016). For fungal VAP, because the Candida species are commonly cultured in respiratory samples, it is rarely an etiology of VAP, rarely causes invasive disease, and it is not recommended to use the routine administration of antifungal therapy when Candida species are found in the pulmonary secretions of mechanical ventilation patients (Kalil et al., 2016). Two groups of risk factors for VAP have been identified, including host-related factors and ventilation-related factors. For example, medical history, gender, age, neurological disorders and comorbidities such as acute respiratory distress syndrome (ARDS), chronic obstructive pulmonary disease (COPD), ulcer disease, organ failure, and immunosuppression are particularly important causes of VAP. In addition, ventilation-related factors usually include duration of the mechanical ventilation, reintubation, absence of subglottic secretion drainage, nasogastric tubes, tracheostomy, intracuff pressure of less than 20 cm H_2_O, and prior intravenous antibiotic use within 90 days. The causative pathogens and risk factors for VAP are listed in Table 1 (Lau et al., 2015; Kalil et al., 2016; Gupta et al., 2017; Schreiber and Shorr, 2017; Timsit et al., 2017a,b; Kidd et al., 2018; Vandana Kalwaje and Rello, 2018).

**Table 1:** The causative pathogens and risk factors for VAP.

Type of pathogens		Risk factors	
		
		Host-related	Intervention-related
Bacterium	*Methicillin-sensitive Staphylococcus aureus MRSA Pseudomonas aeruginosa Acinetobacter baumannii Streptococcus pneumoniae Escherichia coli Klebsiella pneumoniae Anaerobic bacteria Legionella ESBL-PE*	Medical history and underlying illness Male Age > 60 years Prior central nervous system disorder Immunocompromised Acute underlying diseases Emergent surgery Surgical history Organ failure ARDS COPD Burns ECOM Ulcer disease	Peri-operative transfusion of blood products Duration of the mechanical ventilation Reintubation Supine head position in patients receiving enteral nutrition Enteral nutrition Absence of subglottic secretion drainage Intra-hospital transports Continuous sedation, use of paralytic agents Nasogastric tubes Tracheostomy Frequent ventilator circuit changes Intracuff pressure of less than 20 cm H_2_O Prior intravenous antibiotic use within 90 days
Fungus	*Aspergillus Candida*		
Virus	*Influenza Respiratory syncytial virus*		


*VAP, ventilator-associated pneumonia; MRSA, methicillin-resistant Staphylococcal aureus; ESBL-PE, extended-spectrum β-lactamase–producing Enterobacteriaceae; ARDS, acute respiratory distress syndrome; COPD, chronic obstructive pulmonary disease; ECMO, extra-corporeal membrane oxygenation.*

## Strategies for Prevention of VAP

Multiple international guidelines regarding the prevention of VAP are available, and the current preventive strategies for VAP are mainly directed at colonization and aspiration modification (Table 2).

**Table 2:** Strategies for prevention of VAP.

VAP preventive measures
minimize ventilator exposure
intensive oral care
aspiration of subglottic secretions
maintain optimal positioning and encourage mobility
prophylactic probiotics


*VAP, ventilator-associated pneumonia.*

### Minimize Ventilator Exposure

The first choice for lowering VAP risk is minimizing a patient’s exposure to mechanical ventilation (Klompas, 2015; Boltey et al., 2017). The use of non-invasive ventilation approaches is encouraged, such as bi-level positive airway pressure (BiPAP) or continuous positive airway pressure (CPAP). If mechanical ventilation can’t be avoided, work to minimize its duration. Ventilator weaning protocols (for example, daily interruption of sedation and coordination with a spontaneous breathing trial) or evidence-based care bundles can be effective in shortening mechanical ventilation duration.

### Intensive Oral Care

The oral health quickly deteriorates in mechanically ventilated patients, and professional oral hygiene care is believed to help reduce the risk of VAP (Soh et al., 2011). The bacterial load presented in the teeth, gums, tongue, and oral mucosa is different between patients with or without treated with antibiotic therapy and mechanical ventilation (Torres et al., 1993; Shi et al., 2013). Chlorhexidine, a broad-spectrum antiseptic agent, has been shown to reduce the incidence of VAP when used for oral care. Zand et al. (2017) have suggested that oral decontamination with 2% is more effective in the prevention of VAP and reduction of oropharyngeal colonization compared with 0.2% chlorhexidine. A previous meta-analysis shows that oral care with chlorhexidine might be effective in reducing VAP incidence in the adult population when administered at 2% concentration or 4 times/day (Villar et al., 2016). However, Rabello et al. (2018) suggests that administration of chlorhexidine for the prevention of VAP was inconclusive, so further studies are needed to confirm the role of intraoral chlorhexidine in the management of VAP.

### Aspiration of Subglottic Secretions

The core mechanism in the development of VAP is oropharyngeal secretions accumulate above and below the endotracheal cuff, facilitating the drainage into the lower airways, so the subglottic secretion suctioning seems to be a pivotal way to prevent VAP. A recent meta-analysis of 20 randomized controlled trials (RCTs) performed by Mao et al. (2016) suggested that subglottic suctioning reduced the risk for VAP by 45% compared to the control group, and subglottic secretion suction was recommended for preventing VAP and for reducing ventilation length, especially in the population at high risk of early-onset VAP.

### Maintain Optimal Positioning and Encourage Mobility

It has been shown that supine intubated patients have a higher risk of aspiration of gastric contents compared to intubated patients in semi-recumbent position (Keeping the head of the bed elevated between 30 and 45 degrees) (Torres et al., 1992; Orozco-Levi et al., 1995; Elorza Mateos et al., 2011), suggesting that the semi-recumbent patient position might be an effective and easy method to prevent VAP. In addition, although it seems challenging, early mobility might also be important for intubated patients because it results in more ventilator-free days (Boltey et al., 2017; Wutzler et al., 2017).

### Probiotics

Recently, a new strategy in the fight against VAP is the use of probiotics. It is widely believed that probiotic bacteria can decrease the development of VAP through local and systemic actions that improve intestinal barrier function, increase host cell antimicrobial peptides, regulate the composition of the intestinal flora, and reduce overgrowth of pathogenic bacteria and bacterial translocation (Mattar et al., 2002; Mack et al., 2003; Isakow et al., 2007; Kelsall, 2008). Several studies have also suggested that probiotics can be safe and efficacious in the prevention or amelioration of VAP in ICU patients (Gluck and Gebbers, 2003; Morrow et al., 2010; Petrof et al., 2012). We should keep in mind that the side-effects of the probiotics are also existed, such as transfer of antibiotic resistance, toxic effects on the gastrointestinal tract, occurrence of disease sepsis and endocarditis (Ohashi and Ushida, 2009; Wong et al., 2015). The incompatible results between studies may be due to differences in patients and clinical situations, the method of probiotic administration, the criteria used in the diagnosis of VAP and the sample size, or the heterogeneity of the probiotic strains used. Therefore, the current opinion for probiotics is believed that although probiotics are usually considered to be safe and well tolerated, while the wide, well-planned, randomized and multicenter studies are required to verify the efficacy of probiotics against VAP before they can be recommended for routine clinical application.

## Drug Strategies for Treatment of VAP

It is important to note that once VAP is considered likely for a patient, the selection of initial empiric antimicrobials may have great variability among clinical scenarios. However, currently, evidence supporting a standardized approach to the exact initial selection of antimicrobials is lacking. Antibiotic resistance has exponentially increased over the last decade, and the isolation of MDR pathogen has been identified as an independent predictor of initial inadequate antibiotic therapy and increased mortality (Vardakas et al., 2013), and the risk of MDR is based on the local ecological data, previous colonization, and previous antibiotic therapy received by the patients. Moreover, there are lots of attempts to control VAP, such as novel antibacterial agents, inhaled antibiotics and monoclonal antibodies. We summarized the reference approaches adapted from recent recommendations or studies for the management of VAP (Palmer, 2015; Kalil et al., 2016; Gupta et al., 2017; Schreiber and Shorr, 2017; Timsit et al., 2017a; Bassetti et al., 2018; Kidd et al., 2018; Vandana Kalwaje and Rello, 2018), details were shown in Table 3.

**Table 3:** Drug strategies for treatment of VAP.

Category	Strategies	Notes
Intravenous antibiotics		
Without risk factors	Piperacillin-tazobactam 4.5 g IV q6h	–
	Cefepime 2 g IV q8h	–
	Levofloxacin 750 mg IV daily	–
Gram-positive	Vancomycin 15 mg/kg IV q8–12h (consider a loading dose of 25–30 mg/kg × 1 for severe illness)	–
	Linezolid 600 mg IV q12h	–
	Tedizolid 200 mg oral or IV q24h	Phase III for HAP and VAP (NCT02019420)
Gram-negative	Piperacillin-tazobactam 4.5 g IV q6h	–
	Cefepime or ceftazidime 2 g IV q8h	–
	Ceftolozane–tazobactam 3 g IV q8h	Phase III trial for VAP, HAP (NCT02070757)
	Ceftazidime–avibactam 2.5 g IV q8h	Phase III trial for nosocomial pneumonia including VAP (NCT01808092)
	Levofloxacin 750 mg IV daily	–
	Ciprofloxacin 400 mg IV q8h	–
	Imipenem 1g IV q8h	–
	Meropenem 1 g IV q6h	–
	Meropenem–vaborbactam 2 g IV q8h	Phase III trial for the treatment of HAP/VAP (NCT03006679)
	Imipenem–relebactam 500 mg/250–125 mg IV q6h	Phase III for VAP, HAP (NCT02452047); Phase III for VAP, HAP (NCT02493764)
	Aztreonam 2 g IV q8h	–
	Amikacin 15–20 mg/kg IV q24h	–
	Gentamicin 5–7 mg/kg IV q24h	–
	Tobramycin 5–7 mg/kg IV q24h	–
	Plazomicin 15 mg/kg IV q24h	Phase III for BSI, HAP, VAP (NCT01970371)
	Colistin 5 mg/kg IV × 1 (loading dose) followed by 2.5 mg × (1.5 × CrCl + 30) IV q12h (maintenance dose)	–
	Polymyxin B 2.5–3.0 mg/kg/d divided in 2 daily IV doses	–
Inhaled antibiotics	Colistin	Indetermination
	Aminoglycosides (such as sisomycin, gentamicin, amikacin, and tobramycin)	Indetermination


*IV, intravenous; q, every; VAP, ventilator-associated pneumonia; HAP, hospital-acquired pneumonia.*

### Antibiotic

The aim is to choose antibiotics that target specific pathogens of VAP as narrowly as possible, this will ensure optimal treatment while minimizing overtreatment and negative outcomes. The current recommendations for initial empiric antibiotic selection urge the clinician to consider local microbiology patterns because the inappropriate initial antibiotic choice is associated with increased mortality (Moine et al., 2002; Zahar et al., 2011; Timsit et al., 2017a). In addition to local antibiograms, patient-specific risk factors should be used to identify patients at risk for MDR organisms who may necessitate coverage of methicillin-resistant *Staphylococcal aureus* (MRSA), extended-spectrum β-lactamase–producing *Enterobacteriaceae* (ESBL-PE), *Pseudomonas species*, *Klebsiella species*, or *A. baumannii* until susceptibilities are available (Lee et al., 2013; Duszynska et al., 2015; Timsit et al., 2017b). Antibiotic regimens for VAP are to be treated for a short course (7 or 8 days) according to the recommendation, because the available data suggest there is no significant difference between short-course (7 or 8 days) and long-course (10–15 days) antibiotic regimens in regard to mortality, treatment failure, recurrent pneumonia, or duration of mechanical ventilation (Dimopoulos et al., 2013). The longer courses may be appropriate in some circumstances where the patient may have a delayed clinical response. When it comes to combination therapy, a meta-analysis was performed by Aarts et al. (2008) to evaluate the role of combination therapy as empiric treatment for VAP, which was included in 41 trials and 7,015 patients, the results suggest that rates of mortality and treatment failure between monotherapy and combination therapy are similar, while the adverse events are found to be slightly higher in the combination group. Meanwhile, it is critical that to regularly monitor the pattern of MDR organisms in ICU in the management of VAP, and effective national- and state-level monitoring of resistance patterns, antibiotic policy, and draft guidelines are needed to maintain the effectiveness of antimicrobials and for better VAP control (Gupta et al., 2017). In the past decades, lots of new antibiotics against MDR have been approved for the therapeutic intervention of VAP, and other agents are being investigated, such as tedizolid, meropenem–vaborbactam, imipenem–relebactam, ceftolozane–tazobactam, ceftazidime–avibactam, and plazomicin (Jia and Jia, 2016; Bassetti et al., 2018). We believe that these new approved and investigational agents for the treatment of VAP represent promising options to preserve and enhance our antibiotic choices in the future.

### Inhaled Antibiotics

It was observed in some studies that inhalation antibiotics may have beneficial effect in the clinical cure of VAP (Klastersky et al., 1979; Hallal et al., 2007; Lu et al., 2012; Tumbarello et al., 2013; Palmer and Smaldone, 2014). However, other studies showed no statistically significant effect in their primary outcome (Le Conte et al., 2000; Rattanaumpawan et al., 2010; Lu et al., 2011; Niederman et al., 2012; Doshi et al., 2013). A previously published meta-analysis of 6 randomized controlled trials and 6 observational studies performed in VAP shows that the use of inhalation antibiotics is associated with higher rates of clinical cure, while the statistical significance is not reached in the meta-regression analysis (Zampieri et al., 2015). There is a non-significant improvement in clinical cure when using inhalation antibiotics as an adjunctive treatment in VAP besides using systemic antibiotics until now, and more evidence from larger randomized controlled trials (RCTs) is needed to confirm the appropriateness of this adjunctive treatment (Russell et al., 2016).

### Monoclonal Antibodies

At present, the drugs in development for the treatment of VAP can significantly improve the therapeutic options available. The possibility to administer directed therapy with monoclonal antibodies to specific pathogens is an exciting strategy in the fight against widespread resistance of MDR organisms. For example, Que et al. (2014) have reported that the full course of panobacumab, an anti-lipopolysaccharide (LPS) immunoglobulin M monoclonal antibody, adjunctive immunotherapy targeting serotype-specific LPS of *P. aeruginosa* O11 may have improved the clinical outcome of patients presenting with nosocomial pneumonia. Different monoclonal antibodies, including panobacumab (Lazar et al., 2009; Horn et al., 2010), KB001 (Shime et al., 2001; Baer et al., 2009; Francois et al., 2012), and MEDI4893 (Hua et al., 2014, 2015), directed against components of *P. aeruginosa* or *S. aureus* are now being studied for nosocomial respiratory infections. However, most of these studies are in very preliminary stages of development, and it is actually impossible to know what their future role in the therapy of VAP will be. The use of certain monoclonal antibodies directed against some specific serotypes of specific pathogens is an interesting approach to try to reduce antimicrobial resistance.

## Future Directions

In the coming decade, VAP will continue to be a major infection in the ICU. We anticipate the need for better epidemiologic and diagnostic tools that will inform us about the true incidence of these infections and the impact of specific prevention and treatment strategies. For prevention, a VAP care bundle, nursing care and education are recommended; these strategies have been shown to decrease the health care costs and antimicrobials use, length of ICU stay, and the need of mechanical ventilation therapy (Aiken et al., 2014; Okgun Alcan et al., 2016; Nogueira et al., 2017; Jansson et al., 2019). In addition, a recent study has suggested that *N*-acetyl-cysteine (NAC) is safe and effective to prevent and delay the development of VAP (Sharafkhah and Abdolrazaghnejad, 2018). It’s also very important to use new diagnostic methods (such as next-generation sequencing technology, phase contrast X-ray imaging, lung ultrasonography (Bouhemad et al., 2018), and the electronic nose) and/or new biomarkers (such as phosphatidylinositol 3-kinase regulatory subunit and sarcoplasmic reticulum calcium transporting ATPase) (Jamaati et al., 2018), to find the bacteriology and its frequency of MDR pathogens and to guide more accurate and focused initial antibiotic therapy. Moreover, it is very urgent to develop new drugs for MDR pathogens because of increasing antimicrobial resistance. There will also be further exploration of optimized antibiotic pharmacokinetics (PK) and pharmacodynamics (PD), which will allow us to improve the effectiveness of the treatments of pneumonia caused by MDR organisms as well as to achieve a lower rate of adverse effects. We believe that with focus on VAP epidemiology, diagnostic methods, bacteriology, prevention, and therapy, we will see further improvement in the outcomes of our patients.

## Conclusion

Ventilator-associated pneumonia is an important cause of morbidity and mortality in mechanically ventilated patients, and many strategies have been proposed for the prevention and treatment of this disease. Successful prevention of VAP can save on total costs and is possible using a multidisciplinary clinical and administrative approach. In addition, the early appropriate antimicrobial therapy is critical to improving clinical outcomes for patients with VAP. Unfortunately, clinician failure is common, with about 70% of patients receiving inadequate initial empiric therapy for VAP. Some new antibiotics such as tedizolid, meropenem–vaborbactam, imipenem–relebactam, ceftazidime–avibactam, ceftolozane–tazobactam, and plazomicin, are being developed for VAP to combat our increasingly resistant infecting organisms. Meanwhile, some new options and choices for the management of VAP are also being developed, including inhaled antibiotics and monoclonal antibodies. Future studies are necessary to evaluate these therapeutic strategies in the management of VAP. We hope that the present overview contributes to the prevention and control of VAP.

## Author Contributions

ML and XX conceived the general idea. XX and ML wrote the first draft. TH, JL, and ML revised the manuscript. All authors read and approved the final manuscript.

## Conflict of Interest Statement

The authors declare that the research was conducted in the absence of any commercial or financial relationships that could be construed as a potential conflict of interest.
